# Effect of Speaking Valves on Tracheostomy Decannulation

**DOI:** 10.1055/s-0043-1767797

**Published:** 2023-10-06

**Authors:** Bradley W. Eichar, Thomas M. Kaffenberger, Jennifer L. McCoy, Reema K. Padia, Hiren Muzumdar, Allison B. J. Tobey

**Affiliations:** 1 Department of Otolaryngology, University of Pittsburgh School of Medicine, Pittsburgh, PA, United States; 2 Office of Research and Development, Veterans Affairs Pittsburgh Healthcare System, Pittsburgh, PA, United States; 3 Division of Pulmonary Medicine, UPMC Children's Hospital of Pittsburgh, Pittsburgh, PA, United States

**Keywords:** tracheostomy, decannulation, speaking value, chronic lung disease, pediatric

## Abstract

**Introduction** Despite several pediatric tracheostomy decannulation protocols there remains tremendous variability in practice. The effect of tracheostomy capping on decannulation has been studied but the role of speaking valves (SVs) is unknown.

**Objective** Given the positive benefits SVs have on rehabilitation, we hypothesized that SVs would decrease time to tracheostomy decannulation. The purpose of the present study was to evaluate this in a subset of patients with chronic lung disease of prematurity (CLD).

**Methods** A retrospective chart review was performed at a tertiary care children's hospital. A total of 105 patients with tracheostomies and CLD were identified. Data collected included demographics, gestational age, congenital cardiac disease, airway surgeries, granulation tissue excisions, SV and capping trials, tracheitis episodes, and clinic visits. Statistics were performed with logistic and linear regression.

**Results** A total of 75 patients were included. The mean gestational age was 27 weeks (standard deviation [SD] = 3.6) and the average birthweight was 1.1 kg (SD = 0.6). The average age at tracheostomy was 122 days (SD = 63). A total of 70.7% of the patients underwent decannulation and the mean time to decannulation (TTD) was 37 months (SD = 19). A total of 77.3% of the patients had SVs. Those with an SV had a longer TTD compared to those without (52 versus 35 months; *p* = 0.008). Decannulation was increased by 2 months for every increase in the number of hospital presentations for tracheitis ( *p* = 0.011).

**Conclusion** The present study is the first, to our knowledge, to assess the effect of SVs on tracheostomy decannulation in patients with CLD showing a longer TTD when SVs are used.

## Introduction

Over 100,000 tracheotomies are performed every year. Only 5,000 are in children and 50% of these are performed in kids < 1 year old. 1 2 3 These tracheostomies are most commonly performed in children to allow for treatment of chronic lung disease (CLD) of prematurity, defined by patients that require supplemental oxygen at 28 postnatal days or 36 weeks postmenstrual age. 4 It is well known that pediatric tracheostomies present a higher risk than when performed in adults with complication rates between 15 and 19% and 10-year post tracheostomy mortality rates ranging from 9 to 15%. 3 5 6 7 Furthermore, the tracheostomy specific mortality ranges from 0.5 to 5%. 8 Outside of medical complications, there is a significant financial and time burden for caregivers at home that negatively affects the caregiver's quality of life, sleep, and ability to work. 9 Understandably, there is heavy interest in caregivers, providers, patients, and hospitals in improving the decannulation process and decreasing time to decannulation (TTD).

Several pediatric tracheostomy decannulation protocols have been published, yet there is a wide variety of protocols followed by providers. 10 11 12 While the effect of tracheostomy capping on decannulation has been studied, the effect of speaking valves (SVs) is unknown. 13 14 15 Speaking valves allow for vocalization with a tracheostomy tube placement by redirecting airflow through the vocal folds during expiration. The positive effects of SVs are well studied and have been shown to improve quality of life, decrease risk of aspiration, improve swallow physiology, restore upper airway protective reflexes, normalize subglottic airway pressure, and improve gustation and olfaction. 16 17 18 19 20 21 22

In the present study, we aimed to assess the utility and effectiveness of SVs to decrease TTD. To do so, we looked at a select group of complex pediatric patients, those with CLD requiring tracheostomy. In the process, we also assessed several other common diagnoses and clinical factors related to tracheostomies that we hypothesized would change TTD.

## Methods

Institutional review board approval was obtained (STUDY20060017). A retrospective chart review was performed at a tertiary children's hospital and the patients were reviewed from 2002 to 2019. A waiver for consent was obtained. In total, we identified 105 patients who underwent tracheostomy, carried a diagnosis of CLD, and did not have severe neurological disease. Patients were excluded if they were deceased before discharge after tracheostomy placement ( *n* = 12), had tracheostomy tube placement after elective adenoidectomy complicated by respiratory syncytial virus (RSV) and acute respiratory distress syndrome (ARDS) requiring extracorporeal membrane oxygenation (ECMO) ( *n* = 1), were deceased after hospital discharge ( *n* = 7), were lost to follow-up ( *n* = 7), were not discharged on mechanical ventilation ( *n* = 2), and had bilateral vocal fold paralysis ( *n* = 1). We then proceeded to collect variables from both the inpatient and outpatient settings.

Demographic and birth data were collected including weight and gestational age. A history of congenital cardiac disease, cardiac surgeries, and a history of pulmonary hypertension was also collected. Given the known prevalence of airway abnormalities in patients with prolonged endotracheal intubation, we also collected data on airway interventions prior to decannulation including tonsillectomy, balloon dilation, peristomal or suprastomal granulation tissue excision, and laryngotracheal reconstruction (LTR), which were further broken down into single stage and double stage procedures. Single stage involved LTR with tracheostomy decannulation at the same time as opposed to double stage which involves delayed tracheostomy decannulation.

From the outpatient settings, we collected data on the number of visits to pulmonology, to otolaryngology, as well as visits to the emergency department (ED) for tracheostomy-related complications and upper respiratory infections (URI). An episode of tracheitis was noted if it was the discharge diagnosis from the ED. Increased secretions were noted in addition to tracheitis and were not mutually exclusive with tracheitis. Chronic ventilator data was collected, including if the patient was discharged with a ventilator, the start of ventilatory sprints, and when the ventilator was completely weaned. Speaking valve trial data was collected as well as the TTD.

Statistics were performed with logistic and linear regression and the Mann-Whitney U test and the Likelihood Ratio test. Kaplan-Meier survival curves with Log Rank (Mantel-Cox) tests were used to assess statistical differences in TTD. Cox Regression was used for survival curves controlling for variables. Statistical analysis was performed with IBM SPSS Statistics for Windows version 24 (IBM Corp., Armonk, NY, USA) with *p* < 0.05 determining significance. Bonferroni multiple comparison correction was used when appropriate.

## Results

Out of 105 patients identified, 75 met the inclusion criteria and were included in our analysis. The study population included 45 males and 30 females with an average age at tracheostomy of 122 days (SD = 63) ( Table 1 ). The mean gestational age was 27 weeks (SD = 3.6) and the average birth weight was 1.1 kg (SD = 0.6). Few patients were complicated by oligo- or polyhydramnios (4 [5%] versus 3 [4%], respectively). Suprastomal or peristomal granulation tissue excision was performed in 63 patients (84%; M (SD) number of excisions = 2.23 [1.99]) ( Table 2 ). Balloon dilation (BD) was performed in 26 patients (34.7%; M (SD) number of BDs = 1.45 [2.62]). Laryngotracheal reconstruction of any stage was performed in 28 patients (37.3%).

**Table 1:** Patient demographics

	All *n* = 75	Not Decannulated *n * = 22	Decannulated *n* = 53	*p-value*
Male Sex, *n* (%)	45 (60.0%)	11 (50.0%)	34 (64.2%)	0.305
Age at tracheostomy (days), M ± SD; Mdn	122.6 ± 63.5; 116.0	141.0 ± 54.3; 137.5	115.0 ± 65.9; 103.0	0.013
Birthweight (kg), M ± SD; mdn	1.06 ± 0.6; 0.8	1.27 ± 0.7; 1.0	0.96 ± 0.56; 0.8	0.155
Gestational age (weeks), M ± SD; mdn	27.3 ± 3.7; 26.0	28.5 ± 4.4; 27.0	26.8 ± 3.2; 26.0	0.148
Hydramnios, *n* (%)				
Oligo	4 (5.3%)	3 (13.6%)	1 (1.9%)	0.250
Poly	3 (4.0%)	1 (4.5%)	2 (3.8%)	
Follow-up time in years*, M ± SD; mdn	5.4 ± 2.8; 5.1	3.7 ± 2.1; 2.8	6.1 ± 2.8; 6.0	**< 0.001**

Abbreviations: M, mean; Mdn, median; SD, standard deviation.

Bold indicates significance.

Bonferroni multiple comparison correction was used. *P* < 0.008 indicates significance.

**Table 2:** Operative airway interventions overview- Frequency of patients who underwent balloon dilation, tonsillectomy, granulation excision, and laryngotracheal reconstruction (LTR)

	*n* (%)
Granulation excision	63 (84.0)
Balloon dilation	26 (34.7)
Tonsillectomy	16 (21.3)
LTR	28 (37.3)
Single stage	18 (24.0)
Double stage	8 (10.7)
Single stage twice	1 (1.3)
Double stage then single stage	1 (1.3)

Abbreviation: LTR, laryngotracheal reconstruction

**Fig. 1:**
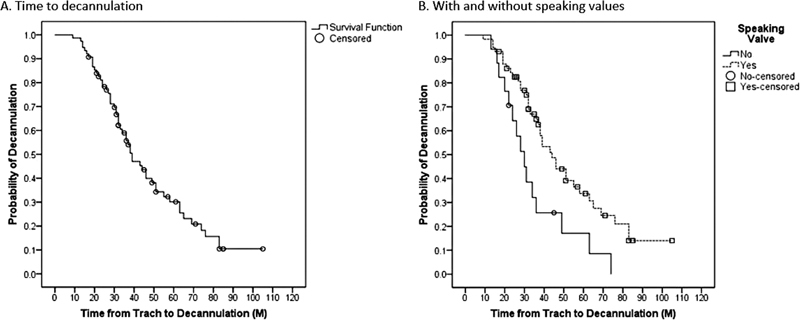
( **A** ) Overall time from tracheostomy to decannulation and ( **B** ) Probability of decannulation with and without speaking valves.

**Fig. 2:**
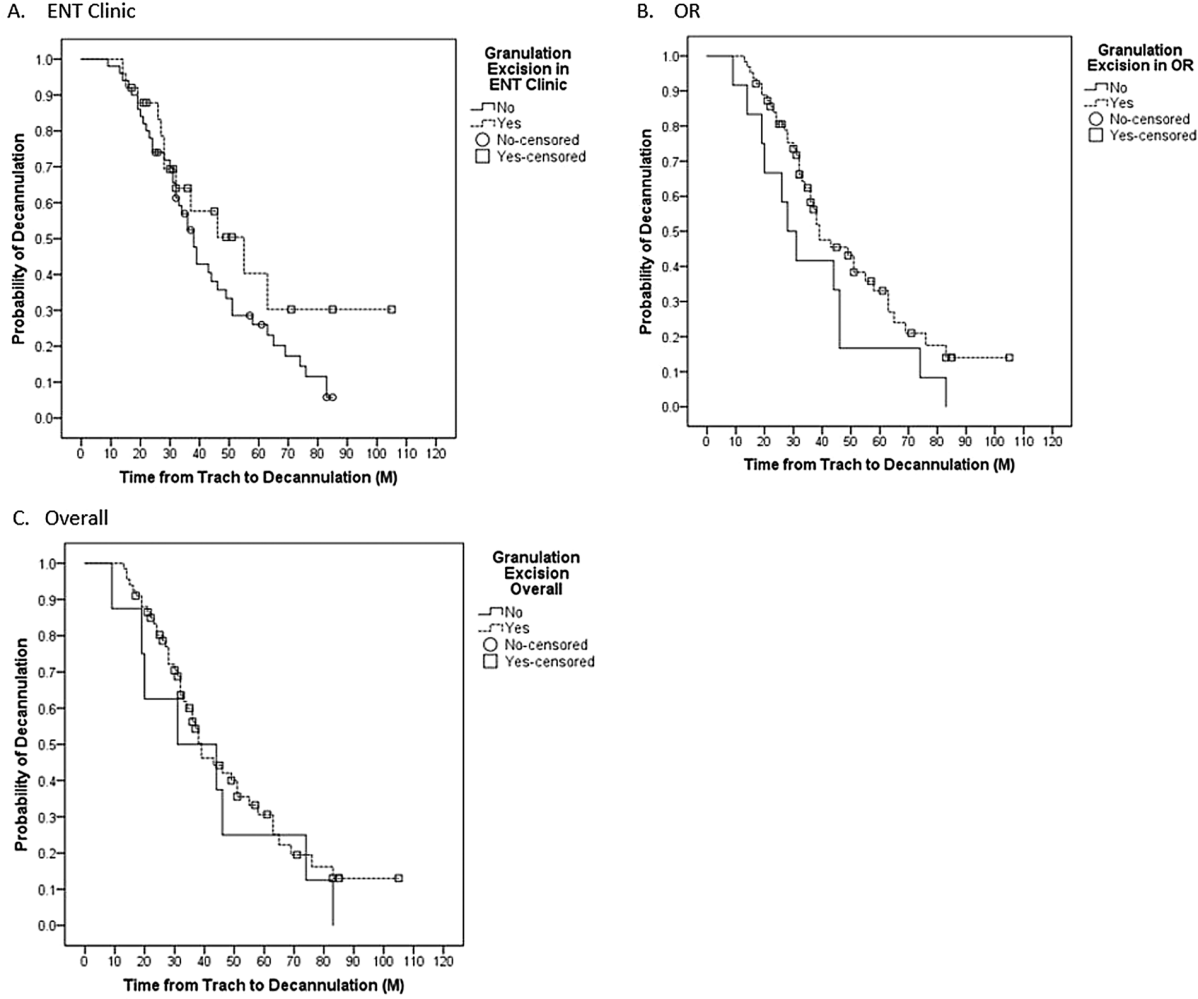
Time to decannulation with the comparison of the probability of granulation tissue in the ( **A** ) Ear, Nose, and Throat (ENT) clinic, ( **B** ) Operating room (OR), and ( **C** ) overall.

**Fig. 3:**
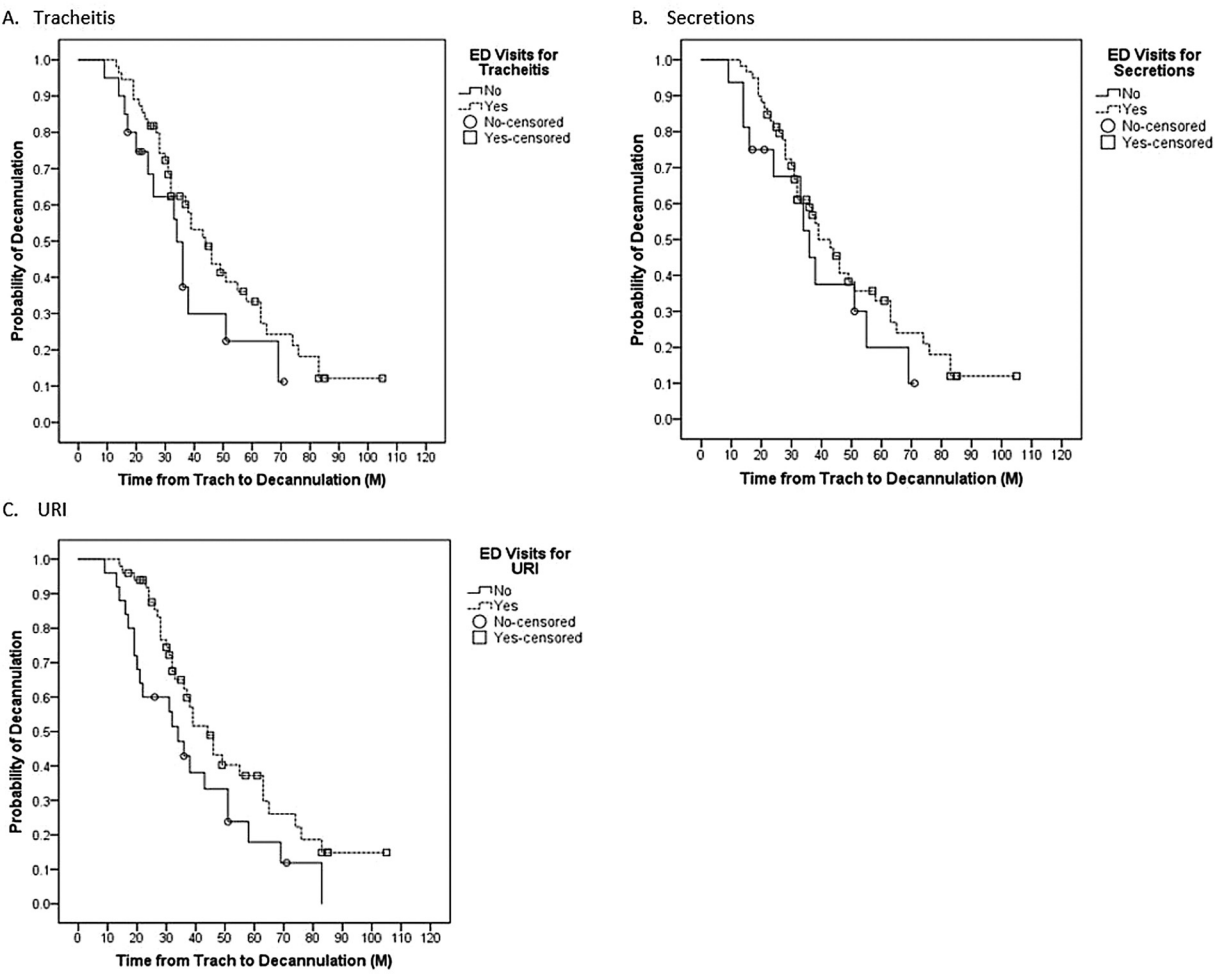
Time to decannulation with the comparison of the probability of emergency department (ED) visits for ( **A** ) tracheitis, ( **B** ) secretions, and ( **C** ) upper respiratory infections (URI).

**Fig. 4:**
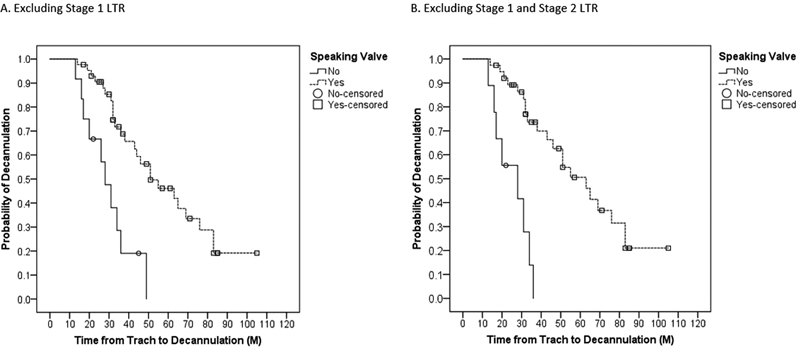
Overall time from tracheostomy to decannulation and with and without speaking valves, ( **A** ) excluding single stage laryngotracheal reconstruction (LTR) ( *n* = 55) and ( **B** ) excluding all LTR ( *n* = 47).

**Fig. 5:**
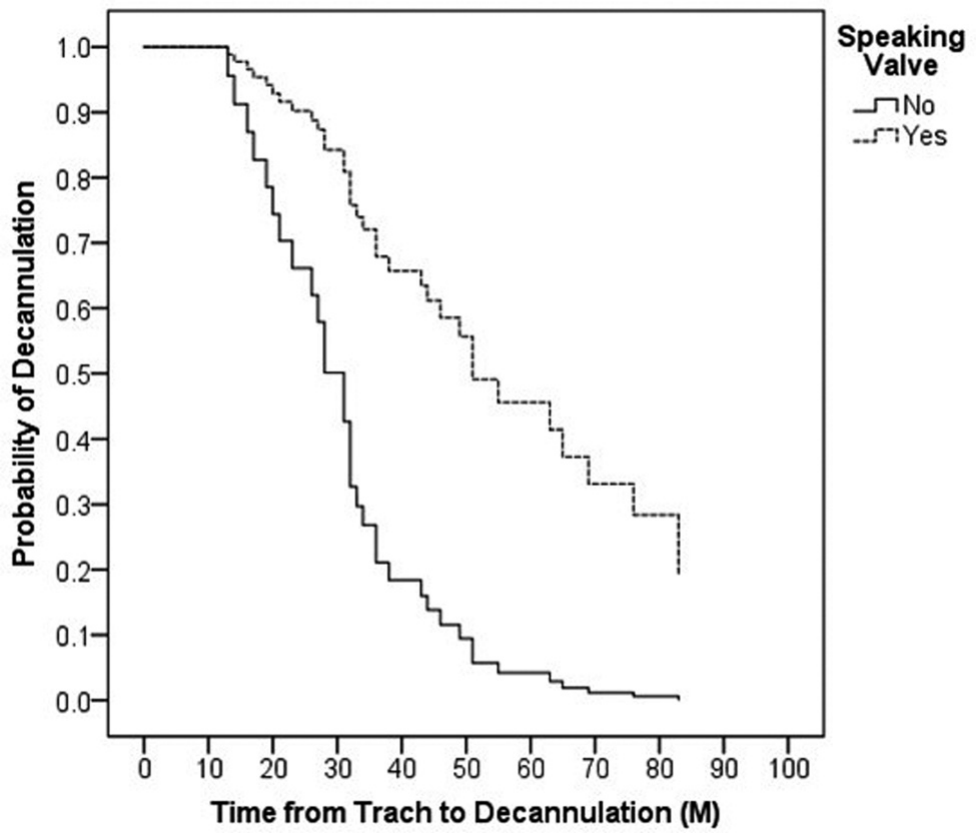
Overall time from tracheostomy to decannulation and with and without speaking valves, when controlling for history of congenital heart disease and excluding single stage laryngotracheal reconstruction (LTR) ( *n* = 55).

Ultimately, 53 patients (70.7%) underwent tracheostomy decannulation and the mean TTD was 37 months (SD = 19) ( Figure 1A ). Prior to decannulation, 58 patients used SVs (77.3%). Those with an SV had a significantly longer TTD compared to those without SVs (Mean 52 versus 35 months, respectively, *p* = 0.008) ( Figure 1B ). Number of granulation tissue excisions in ENT clinic ( *p* = 0.161), the operating room (OR) ( *p* = 0.090), and overall ( *p* = 0.425) did not significantly impact TTD ( Figure 2 ). Emergency department visits for tracheitis ( *p* = 0.137), secretions ( *p* = 0.297), and URIs ( *p* = 0.051) did not significant change TTD ( Figure 3 ). When patients who had a single stage LTR ( *n* = 20) were excluded, those with an SV still had a longer TTD (59 versus 30 months, respectively; *p* < 0.001 ( Figure 4A ). When excluding all patients with an LTR ( *n* = 28), those with an SV also had a longer TTD (62 versus 25 months, *p* < 0.001) ( Figure 4B ). When controlling for a history of congenital heart disease requiring surgery and excluding those with single stage LTR, those with SV use had a higher probability of a longer TTD, *p* = 0.001 ( Figure 5 ).

There was a significant increase in the number of granulation excisions in the ENT clinic in patients who would not be decannulated compared to those who were (M [SD] = 1.45 [2.39] versus 0.25 [0.48], respectively, *p* = 0.001) ( Table 3 ). The number of granulation excisions in the ENT clinic was not correlated with the TTD ( *p* = 0.242). Fifty-five patients (73%) presented to the ED with tracheitis. There was no significant increase in the number of visits for tracheitis in patients who were not decannulated compared with those who eventually were (p= 0.408). There was no difference in the likelihood of being decannulated predicted by the number of ED visits or admissions for tracheitis ( *p* = 0.261). However, there was a significant positive correlation between TTD and number of ED visits or admissions for tracheitis (r = 0.347; *p* = 0.005). Decannulation was increased by 2.12 months for every additional visit to the ED or admission for tracheitis, (b = 2.12; t[51] = 2.64; *p* = 0.011). When Cox regression was performed with significant predictors (SV and ED/admission for tracheitis) as independent variables and TTD as the dependent variable, both SV (p = 0.014) and number of ED visits or admissions for tracheitis ( *p* = 0.015) remained significantly associated with a decreased probability of discharge over time.

**Table 3:** Complications and follow-up between those who were decannulated versus not decannulated

	Decannulated *n* = 53 M ± SD; mdn (range)	Not Decannulated *N* = 22 M ± SD; mdn (range)	*p- value*
# ED visits for tracheitis	2.2 ± 2.8; 1.0 (0-14)	3.1 ± 3.7; 2.0 (0-13)	0.408
# ED visits for URI	1.3 ± 1.5; 1.0 (0-7)	2.1 ± 2.0; 1.0 (0-7)	0.069
# ED for secretions	2.5 ± 3.1; 2.0 (0-18)	4.0 ± 4.2; 3.0 (0-16)	0.182
# Pulmonology clinic with secretions	0.8 ± 1.4; 0.0 (0-7)	0.7 ± 1.4; 0.0 (0-6)	0.709
# ENT visits with secretions	0.9 ± 1.5; 0.0 (0-6)	0.8 ± 1.0; 0.0 (0-3)	1.000
# Granulation excision in surgery	2.1 ± 2.1; 2.0 (1-10)	2.5 ± 1.7; 2.0 (1-6)	0.256
# Granulation excision in ENT	0.3 ± 0.5; 0.0 (0-2)	1.5 ± 2.4; 1.0 (0-10)	**0.001**
# Pulmonology appts	6.4 ± 3.5; 6.0 (0-16)	7.1 ± 5.0; 5.5 (2-21)	0.953
# ENT appts	7.8 ± 5.9; 6.0 (0-28)	8.1 ± 5.4; 6.0 (1-23)	0.505

Abbreviations: ED, emergency department; ENT, Ear, Nose, and Throat; M, mean; Mdn, median; SD, standard deviation; URI, upper respiratory infection.

Bonferroni multiple comparison correction was used. *p* < 0.006 indicates significance.

There were no significant differences in the number of pulmonology and ENT clinic visits and visits to these departments with secretions, number of ED visits with URI and secretions, or the number of granulation excision in the OR between those who were and were not decannulated ( *p* > 0.05) ( Table 3 ).

## Discussion

The primary aim of our study was to assess whether SVs are effective in decreasing TTD in a complex population of pediatric patients with CLD requiring a tracheostomy. While prior studies have assessed tracheostomy capping and the effect of tracheostomy indication, age, and birth maturity on decannulation, 13 14 15 23 here we report the first study, to our knowledge, of SVs on TTD. Factors influencing TTD are of interest given the significant burden tracheostomies place on caregivers and the immense amount of care a child will require though their lifetime. 9 24

Prior studies have reported a wide range of successful decannulation rates between 25 and 75%, which expectedly vary depending upon substantial differences in the indication for tracheostomy and comorbidities of the study population, follow-up, and institutional practices. 24 25 26 27 28 29 Our study limited these variations by selecting tracheostomy patients who had been diagnosed with CLD. However, there are a wide range of patient factors that could have contributed to candidacy for decannulation, such as supraglottic patency and neurologic status. In addition, similar to other studies, the present study is limited by its retrospective nature and the limitations inherent to that study design. Furthermore, our institution has had over 20 pediatric otolaryngologists over the course of this review without any standardized protocol for SV use leading to a wide variety of practice despite our attempts to make this population as uniform as possible. Additionally, our study lacks statistical power to detect some differences; however, we are limited by our patient population. A multicenter study would be optimal to detect differences.

Compared with other decannulation studies, our study fell on the higher end of this range with 70.7% of patients being decannulated and a mean TTD of 37 months (SD = 19), despite representing a premature population with a mean gestational age of 27 weeks (SD = 3.6). The majority of our patients utilized an SV prior to decannulation (77.3%), and contrary to our hypothesis, SVs were associated with a longer TTD compared to those without SVs (52 versus 35 months, respectively, *p* = 0.008). Despite controlling for several prematurity-related pathophysiologic processes including congenital heart diseases and airway surgeries related to subglottic stenosis, this result continued to hold true. However, it must be taken with caution as there is likely a strong component of selection bias where patients with longer TTD and patients that are seen in clinic more frequently are more likely to have an SV trial. In addition, longer planned TTD due to comorbidities may have made providers and caregivers more likely to pursue SV trials to encourage speech and language development while waiting for the child to achieve candidacy for decannulation. It is possible that the slight increase in positive end-expiratory pressure (PEEP) generated by an SV may result in prolonged TTD; however, further studies will be needed to evaluate this. Ultimately, we do not believe that this data should be used to discourage patients or providers from utilizing SVs as this would ignore the psychosocial and developmental benefits provided by these devices.

Although the present study was not designed to demonstrate the benefits of SVs other than decreased TTD, evidence supporting these benefits has been provided in previous pediatric literature. Zabih et al. performed a scoping review of the literature available in 2016. 30 They identified 8 studies reporting verbal communication with SV use. 31 32 33 34 35 36 37 38 More recently, Buswell et al. reported improvements in phonation (new phonation in a previously aphonic child or increase in spontaneous phonation time) in 76% of children with SV. 39 Even in prelingual infants and children with neurologic deficits impacting verbal communication goals, the ability to produce audible crying and nonspecific vocalization can significantly improve patient safety and quality the of life of caregivers. Regarding benefits outside of vocalization, Ongkasuwan et al. reported a reduction in pyriform sinus residue, although no studies have demonstrated a significant reduction in aspiration with SV use. 37 40 Improvement in cough and constipation have also been theorized due to the ability to generate supraglottic pressure and perform Valsalva, respectively, although supporting evidence in children is absent. 37 41 Notably, there is a paucity of studies identifying nonvocalization outcomes as primary or secondary outcome measures in children with SV, especially when compared with the adult literature. This gap highlights the need for future research including both objective outcomes and parent report measures to better define the benefit/risk ratio for SV in children and guide clinical decision-making.

It is also worth noting that there was a significant increase in the number of granulation excisions performed in the ENT clinic in patients who would not be decannulated, although the number of excisions was not correlated with TTD. Similar to the selection bias hypothesized to be occurring in SV placement, patients not able to be decannulated may have been seen more frequently and had increased number of granulation excisions. Furthermore, we found a positive correlation between TTD and the number of ED visits or admissions for tracheitis, with TTD being increased by 2.12 months for every additional visit to the ED or admission for tracheitis. Patients with tracheitis who required a hospital visit likely required more follow-up visits and treatment that further delayed their decannulation. This finding highlights the importance of diagnosing and treating tracheitis early and effectively, as it can have long lasting impacts on time requiring a tracheostomy tube.

## Conclusion

The present study is the first, to our knowledge, to assess the effect of SVs on tracheostomy decannulation in patients with CLD. We show that SVs are associated with longer TTD, even when controlling for patients that required congenital heart surgery or LTR. Speech valves have been shown to improve the rehabilitation process in many ways, and we believe their use should be encouraged. However, the association between SVs and a prolonged decannulation process is something providers should be aware of, as this relationship is further studied.
